# Evaluation of selected interleukins in patients with different gastric neoplasms: a preliminary report

**DOI:** 10.1038/srep14382

**Published:** 2015-10-21

**Authors:** Anna Madej-Michniewicz, Marta Budkowska, Daria Sałata, Barbara Dołęgowska, Teresa Starzyńska, Wojciech Błogowski

**Affiliations:** 1 Department of Gastroenterology, Pomeranian Medical University in Szczecin, Poland; 2 Department of Medical Analytics, Pomeranian Medical University in Szczecin, Poland; 3 Department of Microbiology and Immunological Diagnostics, Pomeranian Medical University in Szczecin, Poland; 4 Department of Internal Medicine, University of Zielona, Góra, Poland

## Abstract

Abnormal interactions between cytokines may be an overlooked mechanism linking the development of different types of gastric neoplasms. In this study a comprehensive analysis of the systemic levels of interleukins (IL-1,IL-6, IL-8,IL-10 and IL-12) was performed in 75 patients with different gastric neoplasms (cancer, gastrointestinal stromal tumors, neuroendocrine neoplasms, lymphomas) and 40 healthy volunteers. Patients with gastric cancer (GC) have significantly higher IL-6 levels, and lower IL-8 and IL-10 concentrations, in comparison to controls and patients with other gastric neoplasms. Analogous results were observed in terms of IL-6/IL-8 and IL-6/IL-10 ratios, whose values were also higher in GC patients. In GC patients no associations were detected between the systemic levels/values of interleukins (ratios) and TNM staging. IL-6, IL-10, IL-6/IL-8 and IL-6/IL-10 ratios appeared to hold diagnostic potential in confirming/excluding the presence of GC. Their sensitivity/specificity in GC detection/exclusion was approximately 54–72%. In conclusion, disturbed systemic biochemical balance in multiple interleukins exists at the earliest stages of and appears to be specific to GC. The interleukin ratios proposed here seem to be more promising indicators of GC in humans than direct systemic levels of interleukins, and probably possess the potential to be applied as a supporting factor for techniques routinely used.

Gastric neoplasms constitute some of the most commonly occurring and heterogenic tumors, which are associated with a high percentage of mortality among affected individuals. In 95% of gastric tumor cases, the presence of gastric cancer is diagnosed. However, within the gastric tissue, several other types of malignancy may arise, including lymphomas, gastrointestinal stromal tumors (GIST), and neuroendocrine neoplasms (NEN). Interestingly, according to recent analyses, the clinical prognosis of patients suffering from these other types of gastric tumors is much more favorable in comparison to most individuals diagnosed with (especially advanced) gastric cancer^1^,^2^,^3^,^4^,^5^,^6^. Unfortunately, despite multiple research efforts being direct towards discovering the molecular, biochemical, and immunological mechanisms responsible for this phenomenon, it remains poorly understood.

For several years, researchers have attempted to identify the exact mechanisms responsible for the formation of neoplasms within the gastric tissue. Thus far, various risk factors have been defined as well as some crucial genetic mutations that may be involved in gastric oncogenesis^7^,^8^,^9^,^10^. Nevertheless, within recent years, much attention has been paid to the biochemical and immunological network of cytokine interactions that may not only contribute to, but also promote, the progression and metastatic spread of these tumors. Several multi-center studies demonstrated that the presence of various polymorphisms in genes coding for IL such as IL-1, IL-6, and/or IL-8 is strongly associated with increased risk of gastric cancer development^11^,^12^,^13^,^14^. Moreover, few clinical cross-sectional studies demonstrated that, in patients with gastric cancer, abnormal systemic levels are detected for some cytokines^15^,^16^,^17^. From the biochemical standpoint, such abnormal balance in IL systemic levels may, in fact, be a common mechanism linking the development of these heterogenetic tumors within the gastric tissue, as the action of various IL may significantly influence and/or orchestrate several molecular processes that are crucial for the successful progression and systemic spread of malignancies^18^,^19^,^20^. However, to date, no comprehensive analysis of potential associations between cytokine balance and development of various gastric tumors can be found in the literature. Moreover, little is known about the potential diagnostic and clinical value derived from the measurement of systemic cytokine levels in patients with different types of gastric tumors.

Therefore, in this study, we performed a comprehensive analysis of the systemic levels of a wide panel of ILs in patients with different histological types of gastric neoplasms. Specifically, we (i) compared cytokine levels among groups of patients and healthy volunteers to determine whether there is evidence of a disturbed biochemical balance in IL profiles in patients with gastric neoplasms and, if so, whether this phenomenon is specific only to gastric cancer or may also be present in patients with other histological types of gastric neoplasms; (ii) verified the potential association between systemic levels of the examined ILs and gastric cancer staging; and (iii) estimated potential clinical benefits that can be derived from measurements of IL systemic levels in patients with lesions detected within the gastric tissue, as novel diagnostic serum markers of gastric cancer in humans. We hypothesized that patients suffering from gastric malignancies would present an abnormal systemic balance for certain ILs and that these would be associated with disease progression. We also posited that IL levels could potentially serve as novel markers to distinguish gastric cancer from other gastric malignancies in humans.

## Results

### Analysis of included individuals

Initial comparison of the anthropometric and laboratory parameters between the groups of recruited individuals revealed no statistically significant differences (Table 1). In our study patients with gastric cancer seemed to have lower BMI values and hemoglobin levels than control individuals, but statistical comparison of these demonstrated values close to statistical significance (p = 0.06 for BMI and p = 0.07 for hemoglobin). Also, no statistically significant differences were observed between patients with gastric cancer and those with other gastric neoplasms.

### Comparison of systemic levels of cytokines in patients with gastric cancer, other types of gastric neoplasms, and control individuals

**Figure 1:**
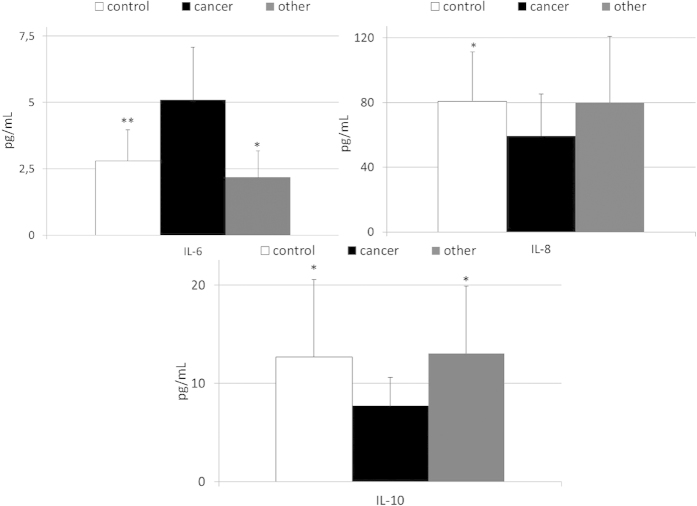
Levels of selected interleukins in patients with gastric cancer, other gastric neoplasms and control individuals together with their statistical comparison (means ± standard deviation) . IL – interleukin *p < 0.01; **p < 0.003 – level of significance (vs cancer group).

The mean systemic concentrations of examined interleukins in patients with gastric cancer, other gastric neoplasms and healthy individuals are depicted in Fig. 1 and Supplementary Figure 1. We found that levels of IL-6 were significantly higher (up to 2 times) in cancer patients than in healthy individuals and patients diagnosed with other types of gastric neoplasms. In contrast, IL-8 and IL-10 concentrations were significantly lower in cancer patients than in healthy controls (Fig. 1). We observed no significant differences in IL-1 and IL-12 levels between healthy individuals and both groups of patients diagnosed with gastric malignancies (Supplementary Figure 1). Interestingly, when we compared levels of examined cytokines between healthy individuals and patients diagnosed with other types of gastric malignancies we did not find any statistically significant differences (Fig. 1 and Supplementary Figure 1).

### Biochemical balance in the concentrations of examined interleukins

**Figure 2:**
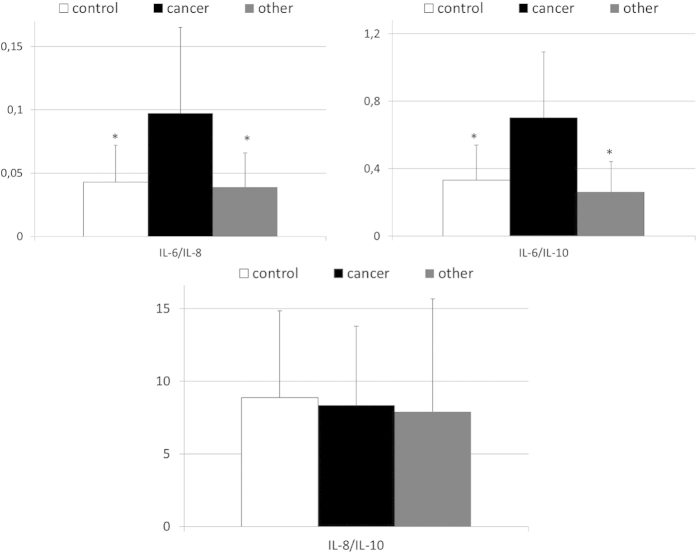
Values of calculated interleukins ratios in patients with gastric cancer, other types of gastric neoplasms and control individuals together with their statistical comparison (means ± standard deviation). IL—interleukin *p < 0.008 – level of significance (vs cancer group).

After we identified the interleukins that differ between gastric cancer patients and individuals from the other examined groups, we calculated relative ratios of their concentrations in order to verify systemic biochemical (dis)balance in their levels among patients with gastric cancer. Indeed, we found that values of IL-6/IL-8 and IL-6/IL-10 ratios were significantly higher in patients with gastric cancer than in both other examined groups, whereas mean IL-8/IL-10 values were comparable between all examined groups (Fig. 2). Also, mean values of above mentioned ratios of interleukins were comparable between healthy individuals and patients diagnosed with other than cancer type of gastric malignancy (Fig. 2).

### Clinical associations between examined cytokines and gastric cancer

**Figure 3:**
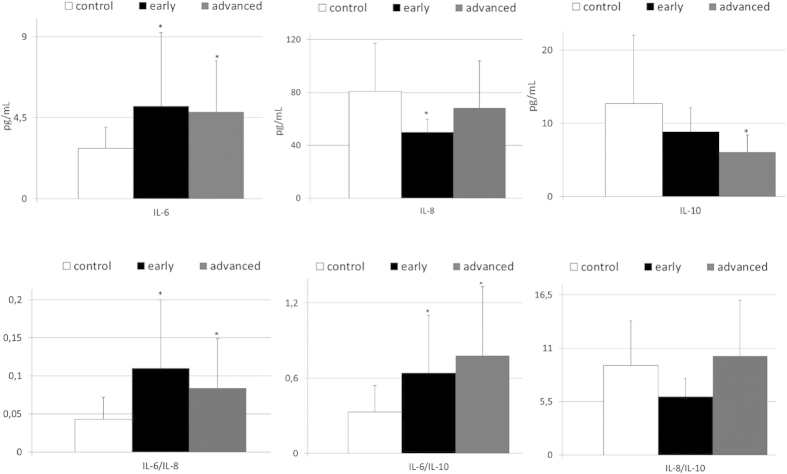
Mean values of selected interleukins’ levels and of calculated interleukins ratios in patients with early and advanced gastric cancer, as well as in control individuals together with their statistical comparison (means ± standard deviation). IL—interleukin *p < 0.004—level of significance (vs control group).

Next, we became interested if the observed alterations in systemic levels of selected interleukins are associated with clinical presentation of gastric cancer among our patients. Therefore, we compared levels of these cytokines in cancer individuals subdivided into two groups – early and advanced gastric cancer. These analyses revealed that in comparison to healthy individuals patients with early gastric cancer have significantly higher mean values of IL-6 levels, IL-6/IL-8 and IL-6/IL-10 ratios, as well as, lower IL-8 concentrations; whereas IL-10 concentrations and values of IL-8/IL-10 ratio were almost reaching statistical significance (p = 0.09 and p = 0.07, respectively; depicted on Fig. 3). Relatively similar results were observed when we compared mean levels of examined interleukins between control individuals and patients with advanced gastric cancer (Fig. 3). Interestingly, no significant differences were stated in terms of IL-1 and IL-12 levels between healthy and both early and advanced cancer patients (Supplementary Figure 2). Furthermore, we analyzed if systemic levels of examined interleukins are associated with clinical staging of gastric cancer, established according to the TNM classification. Using multivariate regression analyses we found that neither plasma levels of analyzed interleukins nor values of interleukins ratios are significantly associated with TNM staging of gastric cancer in our patients (Supplementary Table 1).

**Figure 4:**
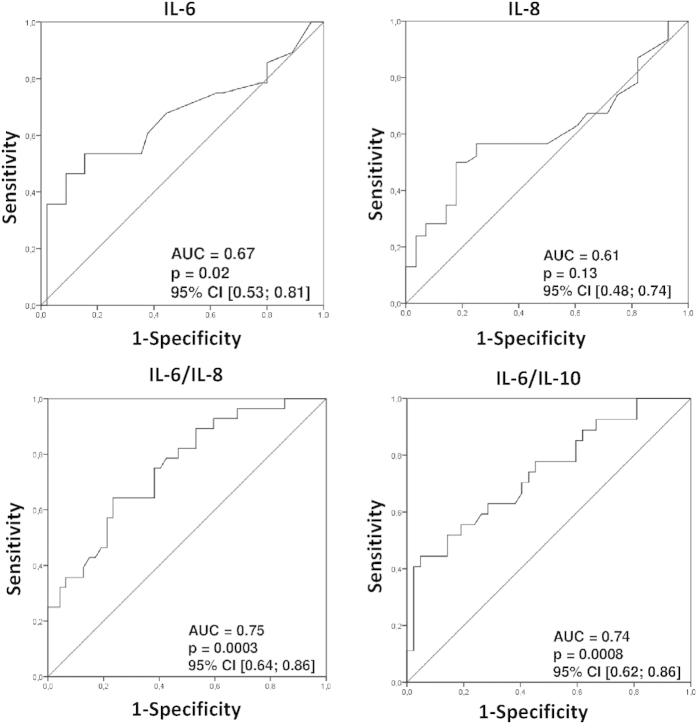
Receiver operating characteristics (ROC) curves of selected interleukins and interleukins (ratios) as indicators of gastric cancer or non-cancerous lesions . Calculated sensitivity (y-axis) is plotted against 1-specificity formula (x-axis) for examined interleukins/cytokines, that is IL-6, IL-8, IL-6/IL-8 and IL-6/L-10 ratios. Precise description of these parameters is presented in Table 2. IL—interleukin AUC – area under curve p – level of significance CI – confidence interval.

Finally, after noting such evident differences in the levels of selected cytokines between patients with gastric cancer and both healthy individuals and those with other types of gastric neoplasms, we decided to preliminarily examine the potential diagnostic value of these selected cytokines for the detection of gastric cancer in humans. To determine whether systemic levels of cytokines could serve as novel makers of gastric cancer, we constructed ROC curves, and determined the approximate AUC values to assess the suitability of these cytokines as potential novel diagnostic markers. Among all examined parameters, only those with a 95% confidence interval (CI) lower bound value that exceeded 0.50 are presented and precisely described (Fig. 4 and Table 2). Our analysis demonstrated that levels of IL-6, as well as, values of IL-6/IL-8 and IL-6/IL-10 ratios are promising potential novel candidate markers for the detection of gastric cancer in humans, whereas IL-8 concentrations might be valuable for its ability to exclude a diagnosis of cancer in patients. Based on our results, we decided to determine suggested diagnostic cut-off values for these cytokines/ratios and preliminarily characterize their estimated sensitivity, specificity, and positive and negative predictive values in our population of individuals analyzed (Table 2). In our study these newly proposed markers proved to be of around 54–72% sensitivity, 54–66% specificity, while their positive and negative predictive values were 47–63% and 50–75%, respectively.

## Discussion

The fact that an intense molecular crosstalk between cytokines and neoplastic cells is crucial for the successful progression of gastric malignancies has been highlighted for many years. Thus far, several experimental studies demonstrated a significant role of various ILs in gastric cancer development (reviewed in detail in^21^,^22^). However, most of these studies only analyzed gastric cancer cells/specimens, but not other types of gastric malignancies. Moreover, these results have never been verified in an actual clinical setting. Therefore, in this study, we comprehensively analyzed systemic levels of several ILs in patients with different types of gastric malignancies. Moreover, we used these data to verify the potential significance of these molecules in both pathogenesis and clinical presentation of gastric tumors in humans.

We found that, among the analyzed ILs, IL-6, IL-8, and IL-10 levels were significantly different among patients with gastric cancer in comparison to both healthy individuals and patients with other types of gastric malignancies. Our results are therefore in line with previously reported observations that were based on the analysis of an Asian population, in which the importance of these cytokines in the development of gastric cancer in humans was highlighted^15^,^16^,^17^. However, we also observed that, in patients with gastric cancer, but not in patients with other types of gastric neoplasms, the biochemical balance in the systemic levels of these ILs was abnormal and “shifted” in a way that seemed to strongly favor the action of IL-6. This phenomenon falls perfectly into the currently proposed molecular scenario, describing the importance of IL-6 in the development and progression of gastric cancer through the promotion of neo-angiogenesis and adhesion of neoplastic cells to the vascular endothelium, thereby supporting the systemic spread of gastric cancer. Moreover, IL-6 supports the survival of cancer cells in both an auto- and paracrine manner^23^,^24^,^25^. Unfortunately, so far the exact “origin” of IL-6 in patients with gastric cancer remains unknown. Some experimental studies demonstrated that this cytokine can be generated by multiple cell types, including neoplastic cells^26^. However, we suspect that this phenomenon does not result from IL-6 generation by cancer cells. In fact, we found that such elevated systemic IL-6 levels (and lower concentrations of other ILs) are also observed in patients with early stage gastric cancer, when the disease is confined to the (sub) mucosal layer of the stomach and does not invade into the muscularis propria or beyond. We believe that this abnormal systemic biochemical balance in ILs may be caused by an altered profile of immune cells in patients with gastric cancer both at the local and systemic levels. Our explanation is based on recent findings, demonstrating that, in patients with early gastric cancer or even with “*in situ*” lesions, systemic and local changes are observed in the profile and direct number of T lymphocytes^27^,^28^,^29^,^30^. In these individuals, much higher infiltration of T regulatory cells (Tregs) is observed within the gastric tissue and a much higher absolute number of circulating Tregs is detected in the peripheral blood. Nevertheless, further clinical and molecular studies are undoubtedly necessary to fully understand this phenomenon in humans.

Many authors demonstrated that among multiple ILs, IL-8 may also play an important role in the pathogenesis of gastric cancer in humans^31^,^32^,^33^. Generation of this cytokine is strongly associated with infection of epithelial cells by cagA + strains of *H. pylori*, and its, IL-8, activity strongly promotes progression and/or systemic spread of gastric cancer. Namely, IL-8 supports neoangiogenesis via stimulation of proliferation and/or survival of endothelial cells. Also due to its effect on inflammatory, stromal and cancer cells it stimulates release of proteases and appropriate growth-modulating factors that enable the cancer cells to not only survive and proliferate, but also to degrade and invade the basal membrane of the stomach and finally form metastasis (reviewed in details in^31^). Surprisingly, while some authors have already highlighted that in patients with gastric cancer overexpression of IL-8 may occur (reviewed in^31^), in our study we did not notice any significant rise in the systemic levels of this cytokine in patients with gastric cancer. This observation may be caused by the fact that generation of IL-8 is seems to be influenced by ethnic origin of analyzed individuals. Our study was based on analysis of the general polish population, which in comparison to other ethnic groups has generally different genetic potential to generate IL-8, and slightly less frequent infections with invasive cagA + *H.pylori* strains are observed among patients with gastric cancer^32^,^34^,^35^,^36^. Unfortunately, in this study we did not routinely examine the presence of *H.pylori* infection and/or its strain in our patients with gastric cancer, so these potential explanations of this phenomenon should be verified in further clinical studies.

Interestingly, in our study, no significant difference was found in IL-1 and IL-12 levels between the examined groups, even though several authors previously proposed a significant role for IL-1 and IL-12 in the pathogenesis of gastric cancer. This discrepancy may result from the examination of different molecular phases of gastric cancer pathogenesis. The presence of various polymorphisms in genes coding for ILs (especially IL-1) has been shown to be strongly associated with increased risk of gastric cancer development in humans^37^,^38^,^39^,^40^,^41^. Both IL-1 and IL-12 play an important role in creating an adequate pro-inflammatory response that is required for the successful elimination of *H. pylori* infection in the stomach^42^,^43^,^44^. The presence or absence of particular polymorphisms in genes coding for ILs seems to determine the individual potential to eliminate *H. pylori* infection (in the context of generation of ILs within gastric tissue). This then defines whether, in a particular patient, a complex cascade of events, initiated by this cardinal risk factor, leading to gastric cancer development occurs. However, in patients that already suffer from gastric cancer, the presence or absence of *H. pylori* infection per se does not seem to exert such important influence^45^,^46^.

It is also important to highlight that systemic levels of the examined ILs in patients with other gastric neoplasms than cancer were identical to those observed in healthy individuals in our study. Therefore, our results suggest that, unlike in epithelial gastric neoplasms, these ILs do not seem to be significantly involved in the pathogenesis of other gastric neoplasms. These results are in agreement with our previous observations regarding pancreatic neoplasms in humans^47^. In our recent report, we demonstrated that several cytokines are strongly associated with both pathogenesis and clinical presentation of pancreatic cancer in humans. However, none of the cytokines analyzed were significantly different between healthy individuals and patients diagnosed with pancreatic neuroendocrine neoplasms or solid pseudopapillary tumors^47^. Therefore, we propose that an abnormal cytokine profile seems to be specific to gastrointestinal cancers. Since (almost) all types of gastrointestinal non-cancerous malignancies may systemically spread and form metastasis, (even though there is no abnormality in the examined cytokine profiles), this observation rises an important question regarding the cardinal and crucial significance of cytokines in the process of metastasis formation in humans. Further experimental and clinical studies are necessary to evaluate this aspect in detail.

Finally, we performed a preliminary analysis of the potential diagnostic value of the analyzed ILs for the detection of gastric cancer and for the discrimination of this disease from other types of gastric neoplasms. In our study, besides analyzing only systemic IL levels, we also calculated ILs ratios, which, in some studies, were proved to be of diagnostic value in patients with different types of malignancies^48^,^49^,^50^,^51^,^52^. We found that systemic levels of only selected ILs and/or IL ratios (IL-6, IL-8, IL-6/IL-8 and IL-6/IL-10) showed potential as diagnostic markers for the detection/discrimination of gastric cancer (with a sensitivity and specificity of approximately 54–72%). Unfortunately, even though our preliminary results seemed promising, at this stage, these markers do not seem suitable for independent decision-making because of their relatively low specificity and because their diagnostic value was determined on the basis of a relatively small sample size. The suggested cut-off and predictive values of these markers should be therefore further verified in cohort studies. In addition, potential influence of *H. pylori* infection on systemic levels of the examined ILs should be verified, and eventually taken into account when interpreting these results.

In summary, our study supported the significance of selected cytokines in the clinical presentation of gastric cancer in humans and demonstrated the existence of a disturbed systemic biochemical balance for several ILs at the earliest stages of gastric cancer development (even in patients with disease limited to the [sub]mucosal layer of the stomach). This imbalance appears to be specific only to this type of gastric malignancy in humans. Moreover, systemic IL levels examined here are not sufficiently sensitive or specific to be used as markers for the detection of gastric cancer. However, the IL coefficients proposed seem to be more promising indicators of gastric cancer in humans and could potentially be used as a supporting factor for routinely used diagnostic techniques.

## Material and Methods

### Ethics statement

This study was performed in accordance with appropriate regulations and guidelines highlighted in the “World Medical Association Declaration of Helsinki – Ethical Principles for Medical Research Involving Human Subjects”. The study protocol was approved by the Institutional Bioethical Committee of the Pomeranian Medical University in Szczecin, and all patients provided written informed consent prior to inclusion in the study.

### Patients and blood samples

A total of 115 individuals with generally good and stable health were included in the study. These patients were divided into groups: a “cancer” group (newly diagnosed gastric cancer, n = 50), an “other malignancies” group (gastrointestinal stromal tumors [GISTs], n = 5; gastric neuroendocrine neoplasms [NENs], n = 12; primary gastric lymphomas, n = 8), and a “control” group (healthy volunteers, n = 40).

The final diagnosis of gastric neoplasm was based on biopsy specimen analysis. In order to establish disease staging, patients underwent ultrasonography, computed tomography and/or endoscopic ultrasonography, as well as chest x-ray examinations. In the “cancer” group 32 patients were diagnosed with intestinal, 12 with diffuse and 6 with mixed type of gastric cancer according to the Lauren’s classification. According to the Tumor Node Metastasis (TNM) classification, 20 patients had stage I gastric cancer, 4 stage II, 4 stage III and in 20 patients the disease presented with metastasis (stage IV). Among our cancer patients 19 patients were diagnosed with early and 29 with advanced gastric cancer, according to the Japanese criteria. In 2 patients we were not able to evaluate the exact stage of the malignancy because they died before any further diagnostic/clinical assessment. Histological analysis revealed following types of gastric cancer in our patients: *adenocarcinoma* (n = 32), *carcinoma mucocellulare* (n = 6), *signet ring cell carcinoma* (n = 3), *carcinoma male differentiatum* (n = 2), and *mixed type* (n = 7).

Among patients with GISTs, in 3 cases stage I low grade tumors were diagnosed, and in 2 patients the tumors were detected in stage II and III with high grade of malignant potential. In all cases GISTs were primarily localized in the fundus of the stomach. In case of patients with NEN lesions, all of the diagnosed tumors were non-functional, and were located in the fundus of the stomach. In 9 patients low grade malignancy tumors were observed (NEN G1), and in 3 cases NEN G2 neoplasms were diagnosed. None of the NEN patients presented any signs of metastasis neither to lymph nodes nor to distant solid organs. Patients suffering from primary gastric lymphomas presented following histological types: diffuse large B-cell lymphoma (n = 4), Burkitt lymphoma (n = 2), small lymphocyte lymphoma (n = 1) and mucus-associated lymphoid tissue lymphoma (n = 1). The general characteristics of the individuals enrolled in the study, together with a statistical comparison of these features between the examined groups, are presented in Table 1.

Peripheral blood samples (8–10 mL) were collected from all included individuals. Samples were processed immediately according to standard laboratory protocols, and plasma was separated, frozen, and stored at –80 °C until further assessment.

### Analysis of systemic levels of cytokines

The systemic concentrations of interleukins (IL-1, IL-6, IL-8, IL-10, IL-12) were measured using commercially available, high-sensitivity ELISA kits (*R&D Systems, Minneapolis, MN, USA*) according to the manufacturer instructions and recommendations.

### Statistical Methods

Analogically as in our previous studies^53^,^54^,^55^,^56^,^57^ the Shapiro–Wilk test was used to determine the distribution of the continuous variables analyzed. The Student’s t-test was used to compare mean parameter values between the examined groups (for normally distributed variables). For variables that were not normally distributed, the values were log transformed. If a normal distribution was then achieved, these transformed variables were also compared using the Student’s t-test. However, if the transformation did not result in a normal distribution, a Mann-Whitney U-test was performed. Correlations between various analyzed parameters were calculated using the Pearson test or Spearman rank test, according to the normality of the distribution. To evaluate the effects of continuous variables on gastric cancer staging and levels of cytokines, multivariate regression analyses were performed using a stepwise selection method. Variables excluded from the initial model were re-entered individually to exclude residual confounding. During development of multivariate regression models, the number of inserted independent variables did not exceed 10% of the total number of analyzed patients. Constructed models were verified using the Akaike information criterion (AIC), and wrongly constructed matrices resulted in rejection of the model. Receiver operating characteristic (ROC) curves were constructed for all parameters analyzed as diagnostic for pancreatic cancer, and the area under each ROC curve (AUC) was calculated. Statistical analysis was performed using SPSS statistical analysis software. P-values less than 0.05 were considered significant.

## Additional Information

**How to cite this article**: Madej-Michniewicz, A. *et al.* Evaluation of selected interleukins in patients with different gastric neoplasms: a preliminary report. *Sci. Rep.* **5**, 14382; doi: 10.1038/srep14382 (2015).

## Supplementary Material

Supplementary Information

## Figures and Tables

**Table 1:** General characteristics of analyzed patients and healthy individuals enrolled in the study (data presented as means ± SD or median [interquartile range]).

Parameter	control	cancer	other
Age (years)	61 ± 6	65 ± 11	60 ± 13
Sex (M-male/F-female)	19-M/21-F	24-M/26-F	5-M/20-F
BMI (kg/m2)	26.18 ± 3.16	24.57 ± 4.11	25.82 ± 5.82
RBC (x10^12^ cells/L)	4.82 ± 0.55	4.26 ± 0.97	4.56 ± 0.40
Hb (g/dL)	14.19 ± 1.75	12.54 ± 2.71	13.22 ± 1.59
Platelets count (x10^9^ cells/L)	220 ± 62	262 ± 87	250 ± 93
WBC count (x10^9^ cells/L)	6.05 ± 1.81	6.45 ± 2.12	6.89 ± 2.35
CRP (mg/L)	2.10 ± 1.04	3.42 [1.10; 12.95]	1.45 [1.33; 4.96]

BMI – body mass index RBC – red blood cells Hb – hemoglobin CRP – C-reactive protein

WBC – white blood cells.

**Table 2:** Diagnostic value of examined cytokines and interleukins ratios to discriminate presence/absence of gastric cancer in our patients.

Parameter	IL-6	IL-8	IL-6/IL-8	IL-6/IL-10
Indication of	cancer	non-cancer	cancer	cancer
Suggested cut-off value	≥2.49 [pg/mL]	≥8.07 [pg/mL]	≥0.042	≥0.34
Sensitivity [%]	68.0	55.4	54.0	72.0
Specificity [%]	63.1	58.0	53.8	66.2
Positive predictive value [%]	58.6	63.2	47.4	62.1
Negative predictive value [%]	71.9	50.0	60.3	75.4

IL – interleukin.
