# Supplementary material for: Evaluation of selected interleukins in patients with different gastric neoplasms: a preliminary report
Source: Sci Rep. 2015 Oct 21;5:14382. doi: 10.1038/srep14382 (PMC4613562; doi:10.1038/srep14382)
Supplement: Supplementary Information [file srep14382-s1.pdf]

Evaluation of selected interleukins in patients with different gastric neoplasms: a preliminary report.

Anna Madej-Michniewicz [1], Marta Budkowska [2], Daria Sałata [2],

*Barbara Dołęgowska [2], Teresa Starzyńska [1], Wojciech Błogowski [3]**

[1] Department of Gastroenterology, [2] Department of Medical Analytics, Pomeranian Medical University in Szczecin, Poland

[3]Department of Public Health, University of Zielona Góra, Poland

Running title: Interleukins and gastric neoplasms.

Article type: Original research article

Correspondence/Reprints to:

Wojciech Błogowski, MD, PhD
Department of Public Health;
ul. Energetyków 2; 65-729 Zielona Góra, Poland;
Tel/fax: (+48) 68 328 73 01;
E-mail: drannab@wp.pl

Supplementary Table 1. Analysis of associations between levels of examined interleukins and clinical presentation of gastric cancer in patients (modelling using multivariate regression analysis).

**Dependent variable**	**Independent variable**	**β**	**R^2**	**p**
*Gastric cancer TNM staging**	IL-6	0.07	0.005	0.74
	IL-8	0.13	0.02	0.53
	IL-10	-0.37	0.13	0.07
	IL-6/IL-8	0.03	0.001	0.87
	IL-6/IL-10	0.18	0.03	0.38
	IL-8/IL-10	0.009	0.01	0.97

β – standardized coefficient in the regression equation

p – level of significance

IL – interleukin

* variable was created by assigning 1, 2, 3 or 4 value to appropriate TNM stage detected in patients with gastric cancer

Supplementary Figure 1. Levels of selected interleukins in patients with gastric cancer, other types of gastric neoplasms and control individuals (means ± standard deviation).

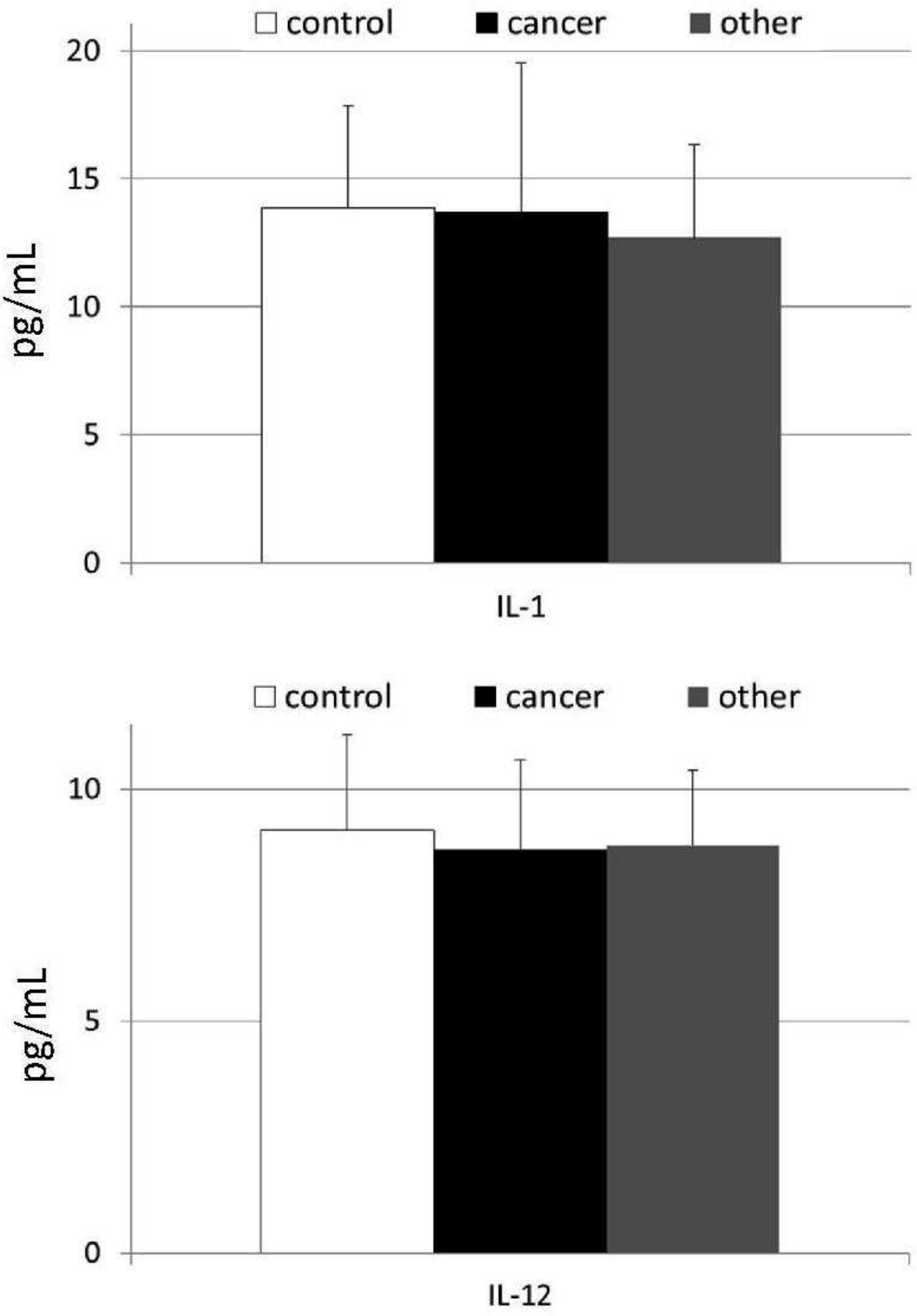

Statistical comparison revealed no significant differences in both IL-1 and IL-12 levels between control individuals, patients with gastric cancer and individuals diagnosed with other types of gastric malignancies (in all cases at least $p > 0.1$).

Supplementary Figure 2. Levels of selected interleukins in healthy controls and patients with gastric cancer, divided into subgroups of early and advanced cancer (means ± standard deviation).

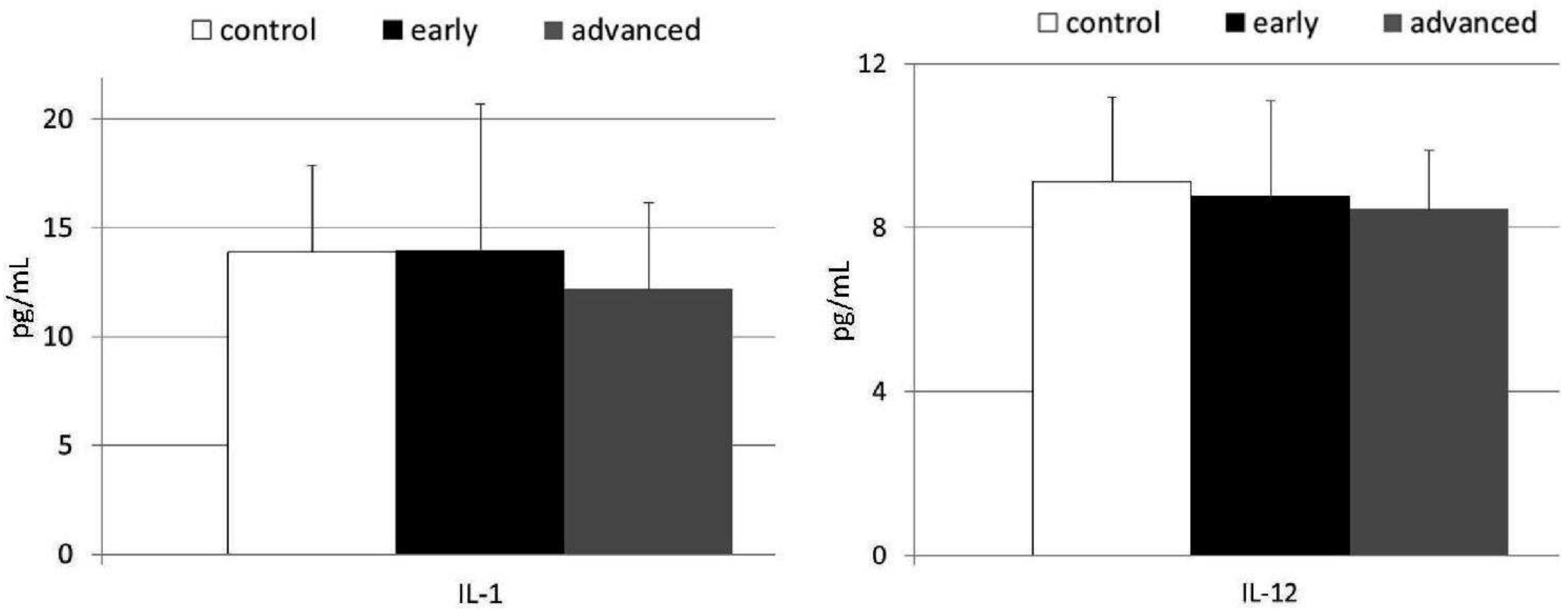

Statistical comparison revealed no significant differences in both IL-1 and IL-12 levels between control individuals, and patients with both early and advanced gastric cancer (in all cases at least $p > 0.2$).
